# A Video-Based DT–SVM School Violence Detecting Algorithm

**DOI:** 10.3390/s20072018

**Published:** 2020-04-03

**Authors:** Liang Ye, Le Wang, Hany Ferdinando, Tapio Seppänen, Esko Alasaarela

**Affiliations:** 1 Department of Information and Communication Engineering, Harbin Institute of Technology, Harbin150080, China; wangle68@huawei.com; 2 OPEM Research Unit, University of Oulu, 90014 Oulu, Finland; Hany.Ferdinando@oulu.fi (H.F.); Esko.Alasaarela@oulu.fi (E.A.); 3 Huawei Beijing Institute, Beijing 100085, China; 4 Department of Electrical Engineering, Petra Christian University, Surabaya 60236, Indonesia; 5 Physiological Signal Analysis Team, University of Oulu, 90014 Oulu, Finland; tapio.seppanen@oulu.fi

**Keywords:** activity recognition, image processing, pattern recognition, school violence detecting

## Abstract

School bullying is a serious problem among teenagers. School violence is one type of school bullying and considered to be the most harmful. As AI (Artificial Intelligence) techniques develop, there are now new methods to detect school violence. This paper proposes a video-based school violence detecting algorithm. This algorithm first detects foreground moving targets via the KNN (K-Nearest Neighbor) method and then preprocesses the detected targets via morphological processing methods. Then, this paper proposes a circumscribed rectangular frame integrating method to optimize the circumscribed rectangular frame of moving targets. Rectangular frame features and optical-flow features were extracted to describe the differences between school violence and daily-life activities. We used the Relief-F and Wrapper algorithms to reduce the feature dimension. SVM (Support Vector Machine) was applied as the classifier, and 5-fold cross validation was performed. The accuracy was 89.6%, and the precision was 94.4%. To further improve the recognition performance, we developed a DT–SVM (Decision Tree–SVM) two-layer classifier. We used boxplots to determine some features of the DT layer that are able to distinguish between typical physical violence and daily-life activities and between typical daily-life activities and physical violence. For the remainder of activities, the SVM layer performed a classification. For this DT–SVM classifier, the accuracy reached 97.6%, and the precision reached 97.2%, thus showing a significant improvement.

## 1. Introduction

In the modern world, the internet has been integrated into people’s daily lives. While online information enriches people’s lives, the probability of children being exposed to violent and gory content also increases in this environment. As a result, school bullying is becoming increasingly more common. School bullying is a violent event that occurs in various forms, such as physical violence, verbal bullying, destroying personal property, etc. Physical violence is considered to be the most harmful to teenagers. According to the “Campus Violence and Bullying” released by UNESCO (United Nations Educational, Scientific and Cultural Organization) in 2017, 32.5% of students all over the world suffer from campus bullying, with a total number of 243 million. Therefore, school bullying prevention is an urgent but timeless topic.

Studies on school bullying prevention have been developed since the 1960s. The methods for school bullying prevention used to be human-driven, i.e., when a school bullying event happened, the bystanders would report the event to the teachers. However, bystanders might fear retaliation from their bullies and thus often do not report the event. As smartphones become increasingly more popular, antibullying applications have been developed for victim use. However, these applications are also human-driven. When school bullying happens, the victim operates the application to send an alarm message, which may enrage the bully to cause further harm.

**Figure 1:**
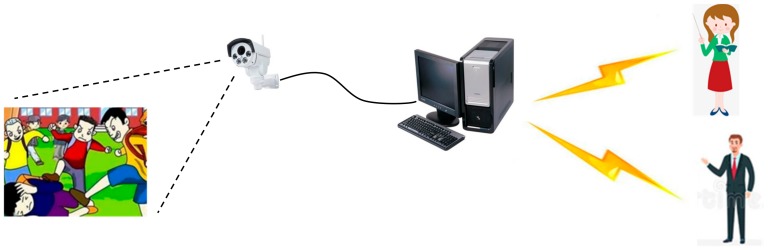
Structure of the violence detecting system.

As artificial intelligence techniques develop, automatic methods are being developed for school bullying detection. In our previous work [1,2,3,4], we used movement sensors to detect physical violence. These movement sensors collected 3D acceleration data and 3D gyro data from the user. Then, the algorithms extracted time-domain features and frequency-domain features from the raw data. Feature selection algorithms, such as Relief-F [5] and Wrapper [6], were used to exclude useless features. PCA (Principal Component Analysis) [7] and LDA (Linear Discriminant Analysis) [8] further decreased the feature dimensionality. We developed several classifiers for different features and different activities, such as FMT (Fuzzy Multi-Thresholds) [1], PKNN (Proportional K-Nearest Neighbor) [2], BPNN (Back Propagation Neural Network) [3], and DT–RBF (Decision Tree–Radial Basis Function) [4], ultimately achieving an average accuracy of 93.7%. These algorithms detect physical violence from the perspective of the victims. In case bullies remove the movement sensors from the victims, this paper proposes an alternative school bullying detection method based on campus surveillance cameras. Figure 1 illustrates the structure of this violence detecting system.

**Figure 2:**
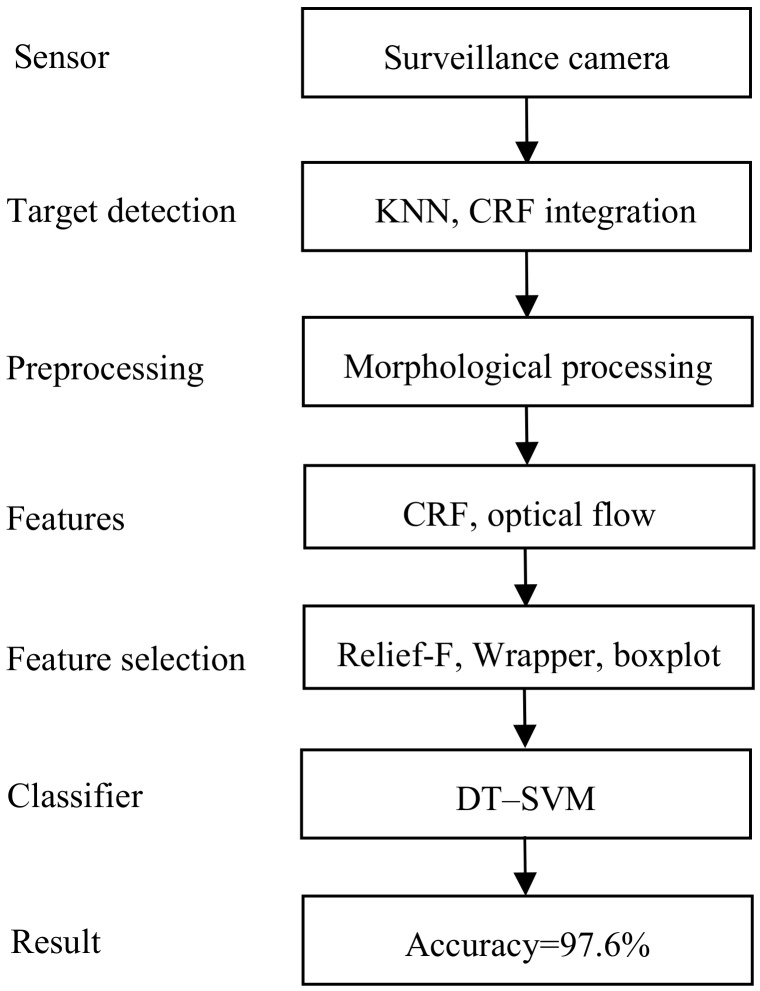
Flow chart of the proposed violence detection method (KNN, K-Nearest Neighbors; CRF, circumscribed rectangular frame; and DT–SVM, Decision Tree–Support Vector Machine).

The surveillance cameras used are conventional security cameras that only capture an image of the campus. All the recognition procedures are performed using a computer. Once a violent event has been detected, alarms are sent to the teacher and/or parents by either short messages or other social media. Figure 2 shows the flow chart of the proposed detection method.

There can be several cameras on campus, each of which monitors a certain area. The camera takes pictures of the monitoring area and first detects whether there are moving targets. If there are moving targets, the KNN (K-Nearest Neighbors) algorithm then extracts the foreground targets. We use morphological processing methods to preprocess the detected targets and propose a circumscribed rectangular frame integrating method to optimize the detected moving target. According to the differences between physical violence and daily-life activities, we extract circumscribed rectangular frame features, such as aspect ratio and optical-flow features, and then reduce the feature dimensionality with Relief-F and Wrapper. By boxplotting, we determined that some features can accurately distinguish physical violence from daily-life activities, so we designed a DT–SVM two-layer classifier. The first layer is a Decision Tree that takes advantage of such features, and the second layer is an SVM that uses the remaining features for classification. According to the simulation results, the accuracy reaches 97.6%, and the precision reaches 97.2%.

The remainder of this paper is organized as follows: Section 2 explores some related works on video-based activity recognition, Section 3 shows the procedures for data gathering and data preprocessing, Section 4 describes the feature extraction and feature selection methods, Section 5 constructs the 2-layer DT–SVM classifier, Section 6 analyzes the simulation results, and Section 7 presents the conclusions.

## 2. Video-Based Activity Recognition

Activity recognition is a popular topic in the areas of artificial intelligence [9,10] and smart cities [11,12]. Most related research focuses on daily-life activity or sports recognition. Sun et al. [13] studied action recognition based on the kinematic representation of video data. The authors proposed a kinematic descriptor named Static and Dynamic feature Velocity (SDEV), which models the changes of both static and dynamic information with time for action recognition. They tested their algorithm on some sport activities and achieved an average accuracy of 89.47% for UCF (University of Central Florida) sports and 87.82% for Olympic sports, respectively. Wang et al. [14] studied action recognition using nonnegative action component representation and sparse basis selection. The authors proposed a context-aware spatial temporal descriptor, action learning units using the graph regularized nonnegative matrix factorization, and a sparse model. For the UCF sports dataset, they achieved an average accuracy of 88.7%. Tu et al. [15] studied Action-Stage Emphasized Spatiotemporal VLAD for Video Action Recognition. The authors proposed an action-stage emphasized spatiotemporal vector of locally aggregated descriptors (Actions-ST-VLAD) method to aggregate informative deep features across the entire video according to adaptive video feature segmentation and adaptive segment feature sampling (AVFS-ASFS). Furthermore, they exploited an RGBF modality to capture motion salient regions in the RGB images corresponding to action activities. For the UCF101 dataset, they achieved an average accuracy of 97.9%, for the HMDB51 (human motion database) dataset they achieved 80.9%, and for activity net they achieved 90.0%.

In recent years, researchers have begun to pay attention to violence detection. A.S. Keçeli et al. [16] studied violent activity detection with the transfer learning method. The authors designed a transfer learning-based violence detector and tested it on three datasets. For violent-flow (videos downloaded from YouTube) they achieved an average accuracy of 80.90%, for hockey they achieved 94.40%, and for movies they achieved 96.50%. Ha et al. [17] studied violence detection for video surveillance systems using irregular motion information. The authors estimated the motion vector using the Combined Local-Global approach with Total Variation (CLG-TV) after target detection, and detected violent events by analyzing the characteristics of the motion vectors generated in the region of the object by using the Motion Co-occurrence Feature (MCF). They used the CAVIAR database but did not give a numerical result for the average accuracy. Tahereh Zarrat Ehsan et al. [18] studied violence detection in indoor surveillance cameras using motion trajectory and a differential histogram of the optical flow. They extracted the motion trajectory and spatiotemporal features and then used an SVM for classification. They also used the CAVIAR dataset and achieved an average accuracy of 91%.

This paper studies the detection of school violence, a kind of physical violence that occurs on campus. The following sections will explain the used dataset and the proposed algorithms in detail.

## 3. Data Gathering and Data Preprocessing

### 3.1. Data Gathering

School violence differs from social violence, such as street fighting or boxing, in the following ways: (1) the victims usually do not resist, (2) no weapons are used, and (3) teenagers are not as strong as adults, so the body motion amplitude of the victims is not as large as that in a social fight between adults. Since there is currently no public school violence dataset, we gathered data by roleplaying school violence and daily-life activities, including campus sports.

**Figure 3:**
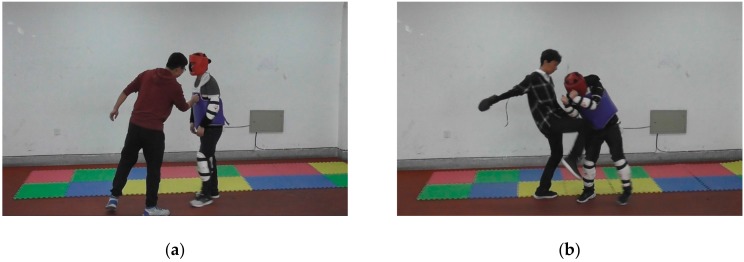

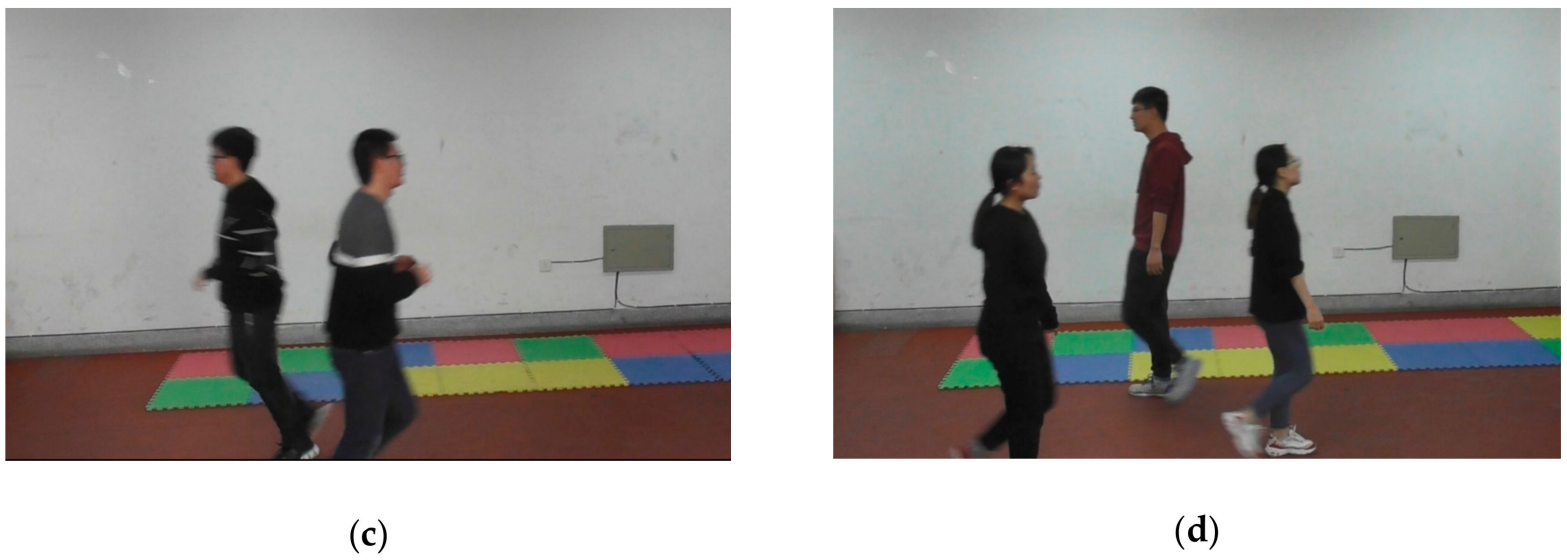
Some examples of school violence and daily-life activities: (**a**) punch; (**b**) kick; (**c**) two people running; and (**d**) three people walking.

Data were gathered by roleplaying school violence and daily-life activities. Several volunteers took turns to act as the bullies and the bullied. The bullied wore protective gear to avoid unexpected injury. Altogether, 24,896 frames were recorded, including 12,448 school violence frames, 9963 daily-life activity frames, and 2485 static frames. Figure 3 shows some examples of school violence and daily-life activities.

### 3.2. Target Detection and Preprocessing

There are several methods for foreground target detection, such as using static differential or frame differential [19]. Static differential is a commonly used method for moving target detection. It first stores a background picture and then compares each input picture with this background. Moving targets are detected according to the difference between the input picture and the background picture. Static differential has a significant disadvantage due to variations in the background, such as changes of light. School violence often happens outdoors, where the light of the scene changes during the day, so the Static Differential is not suitable for this purpose. The frame differential works in a similar way but compares the current frame with the previous one. It is thus more robust against changing light conditions compared with the static differential, but both targets on the current frame and on the last frame will appear in the detected result. One improvement is to multiply the detected result by the current frame, but this will increase the detection errors.

**Figure 4:**
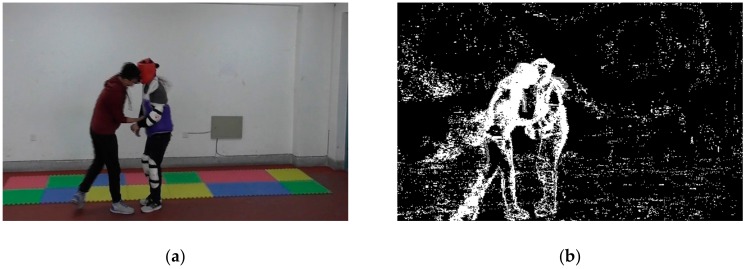

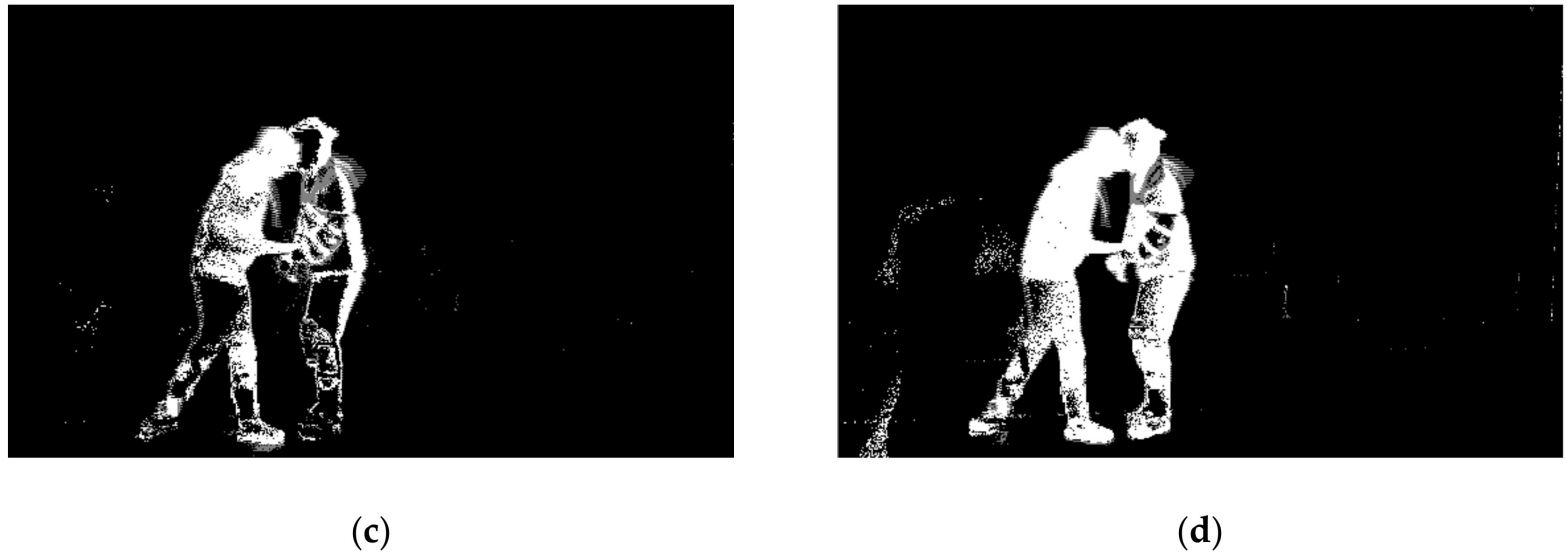
Foreground target extraction with different algorithms: (**a**) original image; (**b**) Geometric Multigrid (GMG) detection; (**c**) Mixture of Gaussians (MOG2) detection; and (**d**) K-Nearest Neighbor (KNN) detection.

As machine learning techniques develop, there are now new methods for foreground target detection, such as KNNs (K-Nearest Neighbors) [20], MOG2 (Mixture of Gaussians) [21], and GMG (Geometric Multigrid) [22]. KNN is usually used as a classifier. KNN classifies a testing sample according to its Euclidean distance to the K nearest training samples in each class. For foreground detection, KNN compares the similarity of the pixels. MOG2 is based on the Gaussian Mixture Model (GMM). MOG2 chooses a proper amount of Gaussian distributions for each pixel, so it offers better adaptions to scene changes. GMG combines static background image estimation and a Bayes segmentation of each pixel. It models the background with previous frames and identifies foreground targets by Bayesian estimation. Since static differential and frame differential have the above-mentioned disadvantages, we selected the KNN, MOG2, and GMG algorithms to extract the foreground targets. The foreground target detection results are given in Figure 4.

Figure 4a is the original picture captured by the camera. Figure 4b is the target detection by GMG. There was too much noise in the picture, and the outline of the person on the left was unclear. Figure 4c is target detection by MOG2. Although it has the least amount of noise among the three algorithms, most pixels of the person on the right side are mistaken as the background. Figure 4d illustrates target detection by KNN. KNN achieves the best balance between foreground information and background noise. Therefore, this paper uses KNN to extract foreground targets. For the background noise, we used a median filter. Then, binarization was performed to enhance the foreground targets.

### 3.3. Morphological Processing

**Figure 5:**
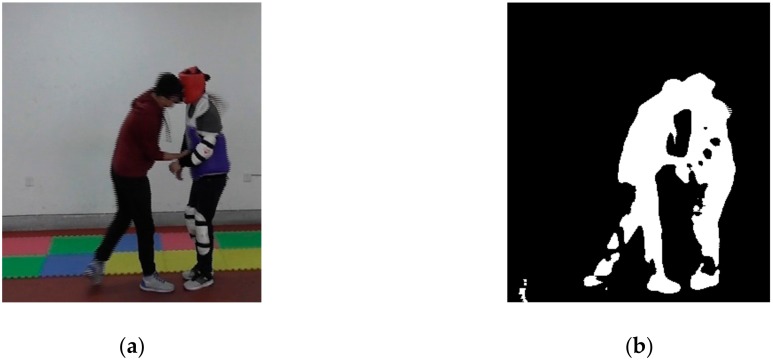

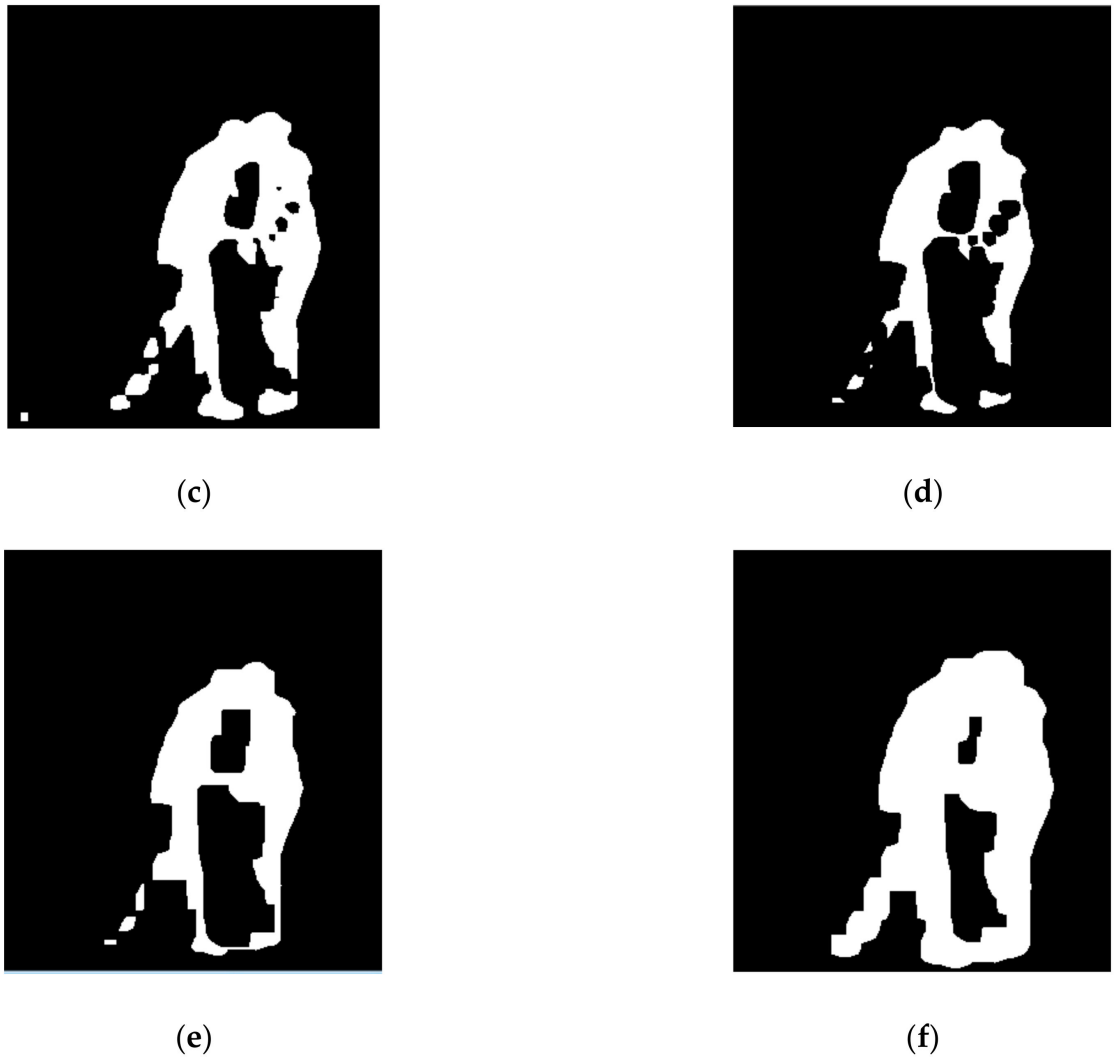
Morphological processing: (**a**) original image; (**b**) target extraction and binarization; (**c**) opening operation; (**d**) erosion; (**e**) closing operation; and (**f**) dilation.

In order to obtain the outlines of the targets, we performed morphological processing. Normally, morphological processing includes dilation, erosion, an opening operation, and a closing operation. Dilation and erosion are the two basic operations in morphological processing. Dilation is usually used to connect separate parts in an image, whereas erosion is usually used to reduce noise. However, in order to avoid changes to the foreground target’s shape, they often work in pairs, which forms the opening operation and closing operation. The opening operation is similar to erosion followed by dilation and is used to remove large background noise points and smooth edges, whereas closing operation is like dilation followed by erosion and is used to connect separate parts in an image and fill in the holes and gaps caused by noise reduction. By choosing the proper morphological processing operations, one can obtain complete foreground targets with the least noise. Figure 5 shows the morphological processing series.

Figure 5a is the original image, and Figure 5b is the image after foreground target detection, median filtering, and binarization. In Figure 5b, there is a block noise at the lower left corner due to the shadow effect. In order to remove this block noise, we first applied an opening operation. As shown in Figure 5c, the area of the block noise was reduced, but the foreground targets were barely affected. Then, erosion was applied to further eliminate this block noise. However, as shown in Figure 5d, the foreground targets were also affected, especially on the legs and the arms. We next performed a closing operation, after which the foot of the person on the right side became connected to his leg, and the holes on his arm were filled, as shown in Figure 5e. Finally, dilation was performed to connect the leg of the person on the left side, as shown in Figure 5f. Although the extracted targets were thicker than real persons, this outline could express the activities of the persons.

### 3.4. Circumscribed Rectangular Frame Integration

Since the outline of the foreground targets is too complex for a computer to analyze, and because a real-time school violence detecting algorithm should have low computational complexity, a circumscribed rectangular frame is usually used instead of the outline itself.

**Figure 6:**
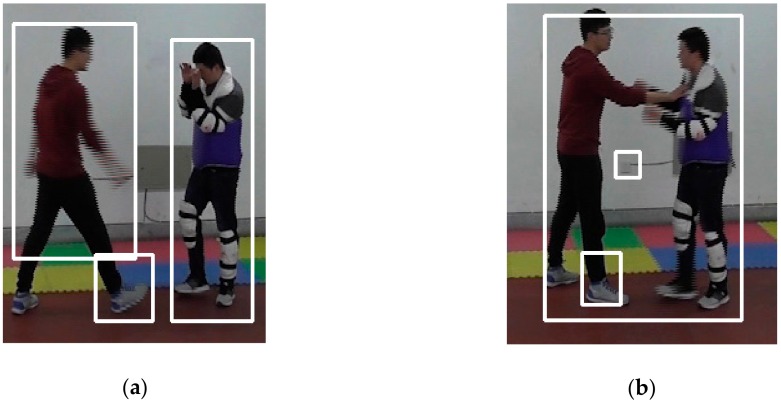
Two examples of unexpected circumscribed rectangular frames: (**a**) one part separated from the whole and (**b**) a redundant circumscribed rectangular frame.

After the foreground target detection mentioned in Section 3.3, we obtained the positions of the foreground target pixels and found the targets in the original image. However, foreground target detection was not always accurate even though morphological processing was performed. Figure 6 gives two examples of unexpected circumscribed rectangular frames.

In Figure 6a, the right foot of the person on the left side was separated from his body during foreground target detection, and the left foot was missing, so there were two circumscribed rectangular frames for this person. The larger frame covered the person from his head to his leg, whereas the smaller one covered the person’s right foot. Figure 6b shows another situation. One foot of the person on the left side was separated from his body during foreground target detection, but the other foot remains connected, so there were also two circumscribed rectangular frames for this person. The larger frame covered him together with the other person because they have touched each other, whereas the smaller frame covered his right foot. There is also a third circumscribed rectangular frame that covered a socket on the wall during foreground target detection due to the shelter when the person moves. In order to obtain accurate circumscribed rectangular frames for the foreground targets, such redundant frames should be fixed.

We propose a circumscribed rectangular frame integrating method. This algorithm is based on collision detection. In a 2-D scene, collision is judged by the circumscribed rectangular frames of the foreground targets. If the distance between any two edges of two circumscribed rectangular frames becomes 0, then a collision occurs. In the proposed algorithm, if two circumscribed rectangular frames collide in horizontal direction and are close to each other in a vertical direction (in other words, the smaller frame is within the regular moving range of the larger one), then the two frames can be integrated as an entire frame. Assume that the coordinates of the two circumscribed rectangular frames are (*x*_1_, *y*_1_, *x*_1_ + *w*_1_, *y*_1_ + *h*_1_) and (*x*_2_, *y*_2_, *x*_2_ + *w*_2_, *y*_2_ + *h*_2_), respectively, where *w_i_* is the width of the frame, *h_i_* is the height, (*x_i_*, *y_i_*) is the lower left corner of the frame, and (*x_i_* + *w_i_*, *y_i_* + *h_i_*) is the upper right corner, *I* = 1, 2. The two circumscribed rectangular frames can be integrated only when

(1) $$\left|\left(x_{1}+\frac{w_{1}}{2}\right)-\left(x_{2}+\frac{w_{2}}{2}\right)\right|<\left|\frac{w_{1}+w_{2}}{2}\right|,$$

and

(2) $$\left|\left(y_{1}+\frac{h_{1}}{2}\right)-\left(y_{2}+\frac{h_{2}}{2}\right)\right|<\left|\frac{h_{1}+h_{2}}{2}\right|+h_{th},$$

where *h_th_* > 0 is a tolerant threshold that represents the position difference of the two frames in a vertical direction.

**Figure 7:**
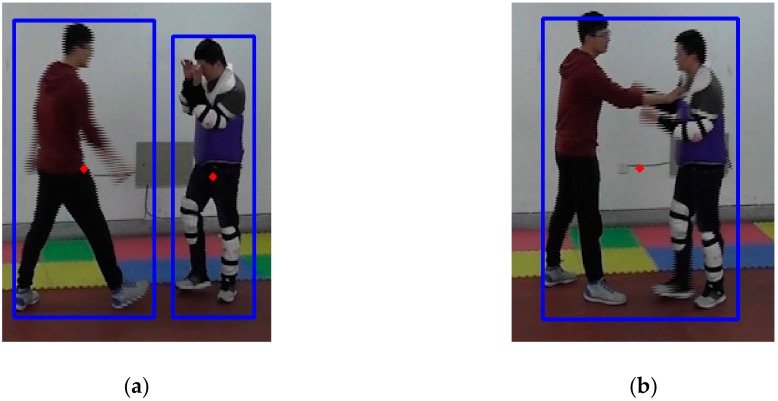
Images after circumscribed rectangular frame integration: (**a**) fixed Figure 4a and (**b**) fixed Figure 4b.

When integrating the two circumscribed rectangular frames, the outermost coordinates of the two frames become the coordinates of the integrated circumscribed rectangular frame, i.e., (min{*x_i_*}, min{*y_i_*}, max{*x_i_* + *w_i_*}, and max{*y_i_* + *h_i_*}), *I* = 1, 2. Figure 7 shows the fixed images according to the circumscribed rectangular frame integration.

Comparing Figure 7a with Figure 6a, the right foot of the person on the left side was reconnected to his body, and the two foreground targets were both detected correctly. Comparing Figure 7b with Figure 6b, the two small redundant circumscribed rectangular frames on the foot and on the socket were integrated into the large frame. There was only one circumscribed rectangular frame in this image because the two persons came together. Thus, the circumscribed rectangular frames were correctly extracted with the proposed algorithm.

## 4. Feature Extraction and Feature Selection

### 4.1. Feature Extraction

Now that the foreground targets (circumscribed rectangular frames) were detected, features were extracted from the detected targets to describe the differences between physical violence and daily-life activities.

#### 4.1.1. Circumscribed Rectangular Frame Features

The following features were extracted from the detected circumscribed rectangular frames.

(1) The number of circumscribed rectangular frames and its variation.
  Firstly, the number of circumscribed rectangular frames could reflect how many persons are there in the monitoring area. If there was only one frame or no frames, then this picture was most likely a non-violent scene. The variation in the number of frames could also indicate the type of activity in the scene. For example, there were two frames in the image at first, and in the next image, the two frames join into one. This probably meant that the two persons had met each other and that the algorithm should judge whether a physical violence was likely to happen. On the contrary, if there was one frame at first that then split into two and never joined again, then it was probably a non-violent scene.
(2) Width of circumscribed rectangular frames and its variation.
  The width of a circumscribed rectangular frame could be used to judge whether the image depicts a single person or multiple persons. If it shows a single person, the image was probably a non-violent scene; otherwise, multiple persons have touched each other, and the algorithm should judge whether it was observing a physically violent event. The variation of width could also indicate the action taking place. For example, when one person hit or pushed another person, the width of their circumscribed rectangular frame (detected as one because they had touched each other) would change.
(3) Height of the circumscribed rectangular frames and its variation.
  The height of a circumscribed rectangular frame could reflect the posture of a person. For example, the height of a frame when a person stands was different than that when he squatted down. The variation of height could reflect the action of a person. When there was more than one circumscribed rectangular frame in a picture, height variation could provide further guidance. For example, if there were two frames in the picture, and one of them suddenly became lower, something had likely happened, which the physical violence detecting algorithm should notice.
(4) Aspect ratio of the circumscribed rectangular frames and its variation.
  These features were a combination of width and the height. The aspect ratio reflected the posture of a person, and its variation reflects the action. When there were multiple frames in a picture, these features could be used to judge whether a physically violent event had happened.
(5) Area of circumscribed rectangular frames and its variation.
  These features could be used to judge the number of persons in one circumscribed rectangular frame and exclude the undesired moving targets. In a physical violence scene, the actions were often intense, and the area of the circumscribed rectangular frame varied significantly and frequently; thus, these features could indicate violence in the algorithm.
(6) Centroid distance of the circumscribed rectangular frames and its variation.
  In a binary image after foreground target detection, the centroid could be considered as the center of the target. Then centroid distance of the circumscribed rectangular frames represents the distance between two persons. When the centroid distance was large, the two persons were relatively far away from each other; when the centroid distance was small, the two persons were close to each other and something may happen between them.
(7) Area of detected targets and its variation.
  These features were extracted not from the circumscribed rectangular frames but from the detected targets after morphological processing, as in Figure 3f.

When there was more than one target (circumscribed rectangular frames) in an image, extract the maximum value of the features, e.g., the maximum width of the circumscribed rectangular frame. However, the maximum area and the sum of the areas were both extracted. Since these features were affected by the distance between the camera and the observed persons, this work did not take distant persons into consideration.

Clearly, there should be a logical relationship between the current frame and the previous frame. Therefore, besides the above-mentioned features extracted directly from the circumscribed rectangular frames, we also defined some states for the circumscribed rectangular frames.

Assume that *Area_max_* is the maximum area of the detected targets in the image, *Area_min_* is the minimum area of the detected targets in the image, *Width_max_* is the maximum width of the circumscribed rectangular frames, *AreaTh_low_* is the lower threshold of a single person, *NonHu_th_* is the threshold of a non-human, *AreaTh_up_* is the upper threshold of a single person, *MulPer*1*_th_* is the threshold of multiple persons, *MulPer*2*_th_* is another threshold of multiple persons, *Cntrd_max_* is the maximum centroid distance of the circumscribed rectangular frames, and *Cntrd_th_* is the threshold of the centroid distance. The difference between *MulPer*1*_th_* and *MulPer*2*_th_* is that *MulPer*1*_th_* is used to judge the number of persons when there is only one circumscribed rectangular frame, whereas *MulPer*2*_th_* is used when there is more than one circumscribed rectangular frame.

We define the states of the images as follows:

(1) When there is one circumscribed rectangular frame in the image,
  a) If *Area_max_* < *AreaTh_low_*, then this image contains only one target, and is marked as State 1;
  b) If *AreaTh_low_* ≤ *Area_max_* ≤ *AreaTh_up_*, the number of targets in this image is uncertain, and this image is marked as State 3;
  c) If *Area_max_* > *AreaTh_up_*, then this image contains multiple targets and is marked as State 4.
(2) When there is more than one circumscribed rectangular frame in the image,
  a) If *Cntrd_max_* > *Cntrd_th_*, then the targets in the image are far away from each other, and this image is marked as State 6;
  b) If *Cntrd_max_* ≤ *Cntrd_th_*, and *Area_max_* ≥ *MulPer*2*_th_*, then the situation in this image is uncertain, and this image is marked as State 3;
  c) If *Cntrd_max_* ≤ *Cntrd_th_*, *Area_min_* < *AreaTh_low_*, and the number of circumscribed rectangular frames is two, then the smaller frame is not a human, and this image is marked as State 1;
  d) If none of the above conditions is met, then the image is marked as State 2.
(3) Moreover, taking the state of the previous frame (image) into consideration,
  a) If the state of the previous image is State 3 or State 4, *Area_max_* > *MulPer*1*_th_*, the number of targets in the image is 2, and *Cntrd_max_* < *Cntrd_th_*, this likely means that two people met but quickly separated, and this image is marked as State 5;
  b) If the state of the previous image is State 4 or State 5, and the number of circumscribed rectangular frames is more than 1,
    i) If *Cntrd_max_* > *Cntrd_th_*, then the image is marked as State 6;
    ii) If *Cntrd_max_* ≤ *Cntrd_th_*, and *Area_max_* ≥ *MulPer*2_th_, the state of the targets in the image is uncertain, and the image is marked as State 3;
    iii) If *Cntrd_max_* ≤ *Cntrd_th_*, *Area_min_* < *AreaTh_low_*, and the number of circumscribed rectangular frames is two, then one of the detected targets is not a human, and the image is marked as State 1;
    iv) If none of the conditions above are met, the image is marked as State 5.

**Figure 8:**
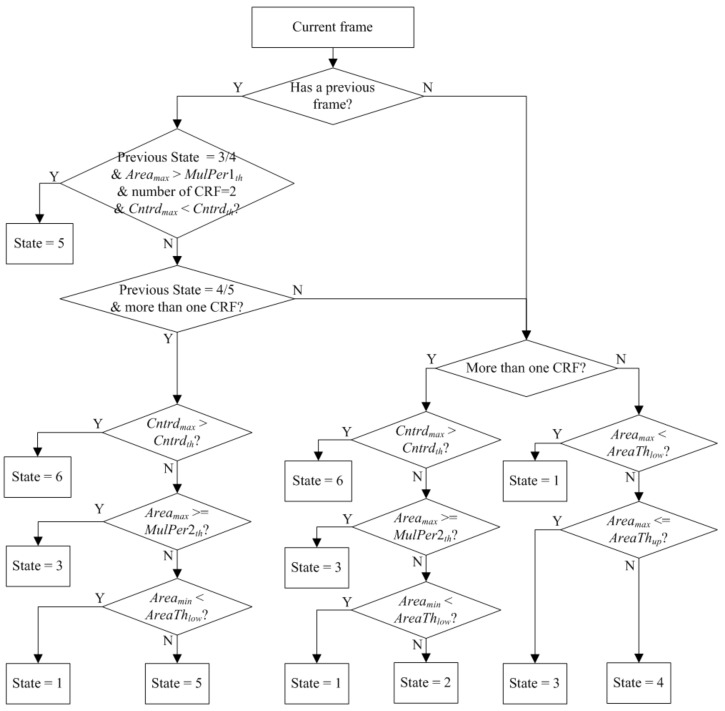
Flowchart of the state definition (CRF, circumscribed rectangular frame).

Figure 8 illustrates the state definition flowchart. These features are very intuitive to judge the movement of the targets, so we designed a Decision Tree classifier for them to determine some typical activities. The details are described in Section 5.

#### 4.1.2. Optical Flow Features

Optical flow is a commonly used method for moving target analysis. An object is represented by pixels in an image, and when the object moves, the luminance of the pixels varies. The variation of luminance is called the optical flow. Optical flow can reflect the movement of an object.

There are two main types of optical flow: dense optical flow and sparse optical flow. Dense optical flow performs image registration with each pixel on the image and calculates the offsets for all the pixels, so it has a high computational cost. Since this work is intended for real time applications, dense optical flow is unsuitable. On the other hand, sparse optical flow performs image registration with sparse points on the image. Given some points (usually corner points) on the reference image, sparse optical flow searches for the corresponding points on the sample image. Assume that *P*(*x*, *y*) is a corner point on reference image *R*, and *Q*(*x*+*u*, *y*+*v*) is the corresponding point on sample image *S*, where *u* and *v* are the offsets in the x-axis and y-axis, respectively. If all the pixels in a small rectangular box centered at *P* are the same with those in the same rectangular box centered at *Q*, then *Q* matches *P*. However, there is noise in actual images, so the pixels in the two boxes cannot be the same. In this case, take the point with the smallest difference as the reference point:

(3) $$\begin{array}{ll} P(x,y) & =\arg\underset{u,v}{\min}E(u,v)\\ & =\sum_{(x,y)\in w}\left|R(x,y)-S(x+u,y+v)\right| \end{array}$$

where *w* is the rectangular box centered at *P*. Equation (3) can be solved by an image pyramid. We extracted the maximum, the 10th maximum [23], and the mean of the optical flow amplitude as the features.

### 4.2. Z-Score Normalization

If a classification model does not have the characteristic of scaling invariance, features of different magnitudes will cause the model parameters to be dominated by larger or smaller data, and thus the training procedure will be affected negatively. When comparing the contributions of features of different magnitudes to classification, normalization is usually necessary.

In this work, we use the Z-Score normalization algorithm, which is calculated as

(4) $$x\prime =\frac{x-\mu }{\sigma }.$$

Z-Score normalization is very simple and thus has a low computational cost. After Z-Score normalization, the training procedure can avoid the problem of instability in numerical calculations due to unbalanced weights.

### 4.3. Feature Selection

Since not all the extracted features can contribute to classification, feature selection is usually a necessary procedure in pattern recognition. We applied different feature selection algorithms in different phases of activity recognition.

#### 4.3.1. Relief-F

Relief-F is used immediately after the image features are extracted. Relief-F is a Filter type algorithm developed from Relief. It takes the *k* nearest samples into consideration instead of only one and supports multiclass classification. Relief-F gives features different weights based on the relevance of each feature and category. The correlation between the features and categories in the Relief-F algorithm is based on the ability of the features to distinguish between close-range samples. Features with weights smaller than a given threshold are removed. In this paper, we used the improved Relief-F proposed in our previous work [4], which further reduced feature redundancy.

We applied Relief-F to the circumscribed rectangular frame (and detected target) features, as well as the optical flow features extracted in Section 3, and removed the features with non-positive weights. Finally, we obtained 14 helpful features: maximum width, maximum width variation, maximum height, maximum height variation, maximum area, maximum area variation, maximum aspect ratio, maximum aspect ratio variation, maximum centroid distance, maximum centroid distance variation of the circumscribed rectangular frame, sum of areas, maximum area, state of the detected targets, and the mean of optical flow.

There features are later used to select the best kernel for the SVM classifier.

#### 4.3.2. Wrapper

Wrapper was used after we determined the kernel function of the SVM because Wrapper needs the classifier to evaluate the contribution of the features. Wrapper can be performed either forwards or backwards or in other combinations (e.g., forwards–backwards and backwards–forwards). Since there are already 14 features in the feature set, we used the backwards–forwards Wrapper. For clarity, we numbered the features as shown in Table 1.

Firstly, a “backwards” procedure was performed. We removed each feature from the feature set and observed the recognition accuracies, beginning with Feature (1). Table 2 shows the accuracy variation during the “backwards” procedure.

After the “backwards” procedure, four features were removed, namely (8), (9), (12), and (14). Since different feature combination can have different effects, in order to avoid removing features by mistake, a “forwards” procedure was performed. Table 3 shows the accuracy variation during the “forwards” procedure.

In the “forwards” procedure, none of the removed four features could improve the accuracy, so these four features were removed from the feature set. After Wrapper feature selection, the accuracy increased from 89.15% to 89.62%. Since the feature dimension after feature selection was not high, we did not perform further dimension reduction procedures. After Relief-F and Wrapper feature selection, all the remaining features contributed positively to classification; further dimensional reduction would lose useful information and thus decrease recognition accuracy. 

#### 4.3.3. Boxplot

A boxplot was used to find the proper features and proper thresholds to design the Decision Tree. As mentioned in Section 3, some features could distinguish typical activities from other activities. Figure 8 shows two examples of such features, namely the sum of the areas of the detected targets and the maximum areas of the detected targets.

**Figure 9:**
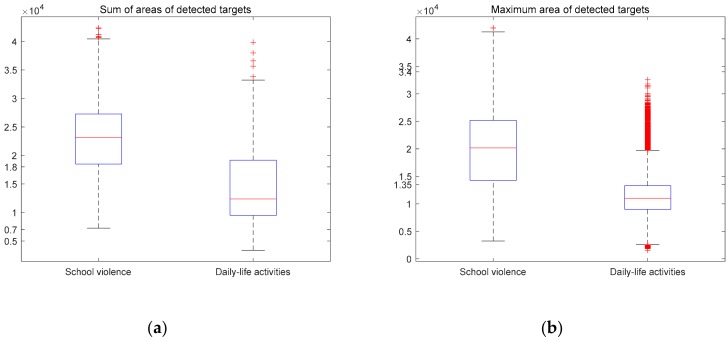
Two examples of boxplots: (**a**) the sum of the areas of the detected targets and (**b**) the maximum areas of the detected targets.

Figure 9a shows the distributions of the sum of the areas of the detected targets for school violence and daily-life activities. The value of 1.8 × 10^4^ is the threshold separating 75% of school violence from 75% of daily-life activities. However, this threshold is good for SVM but imperfect for DT. The value of 0.7 × 10^4^ is another available threshold. Although it cannot distinguish most activities, it can accurately separate a few daily-life activities from school violence because there is no school violence distribution under this threshold. Figure 9b shows the distributions of the maximum areas of the detected targets of school violence and daily-life activities. 1.35 × 10^4^ is a possible threshold that separates 75% of school violence from 75% of daily-life activities. Similarly, this threshold is also good for SVM but not perfect for DT. The value 3.4 × 10^4^ is another threshold. There is no daily-life activity sample distribution above this threshold, so it is possible to separate some typical school violence samples from daily-life activities.

## 5. DT–SVM School Violence Detecting Algorithm

### 5.1. SVM Classification

Since the purpose of this work is 2-class classification (i.e., physical bullying and daily-life activities), we chose SVM as the classifier. SVM has proven to be a good choice for 2-class classification. Moreover, the aim of this work was to design a real-time violence detecting system, so the computational cost of the detecting algorithm had to be as low as possible. Compared with other machine learning methods, such as ANN (Artificial Neural Network), and deep learning methods, SVM has a lower computational cost. SVM provides several kernel functions to fit different data distributions, namely linear kernel functions, polynomial kernel functions, radial basis functions (RBF), and sigmoid kernel functions.

We tested the classification performance of the four kernel functions. The input is the 14 features selected by Relief-F after Z-Score normalization. Five-fold cross validation was used, and the simulation results are given in Table 4.

It can be seen from Table 4 that RBF offers the best performance, so we chose RBF as the kernel function in the following work.

### 5.2. DT–SVM Classification

As mentioned in Section 4, some features can accurately distinguish different activities from each other, so we designed a Decision Tree for these features. Compared with other classifiers, the Decision Tree has several advantages, such as a low computational cost and robustness against noise. The main task in designing a Decision Tree is to find their proper features and proper thresholds.

Figure 8 in Section 4 shows two examples of how to find the proper features and their corresponding thresholds. We repeated a similar procedure for the other possible features and thresholds. Table 5 shows the selected features and their corresponding thresholds.

In order to be more generic, the thresholds were given a margin of error. For example, in Figure 9a, the exact threshold to separate typical daily-life activities from school violence was 7000 pixels^2^, but to be more general, we set the threshold in DT to be 6000 pixels^2^. The same was done for the other thresholds.

The DT and SVM work in a 2-layer mode. If DT can exactly determine the tested activity (if one of the conditions in Table 5 is met), then DT determines the classification result; otherwise, the SVM determines the classification result.

## 6. Experimental Results

In this experiment, we altogether captured 24,896 frames of activities, including 12,448 school violence frames, 9963 daily-life activity frames, and 2485 static frames. After feature selection, 10 features were used for SVM classification: maximum width, maximum width variation, maximum height, maximum height variation, maximum area, maximum area variation, maximum aspect ratio, the maximum centroid distance variation of circumscribed rectangular frames, and the sum of areas and state of the detected targets. These features and their corresponding thresholds used for DT are given in Section 5.2.

We define school violence as positive, and daily-life activity as negative, so TP (True Positive) means that school violence is recognized as school violence, FP (False Positive) means that daily-life activity is recognized as school violence (also known as a false alarm), TN (True Negative) means that daily-life activity is recognized as a daily-life activity, and FN (False Negative) means that school violence is recognized as a daily-life activity (also known as a missing alarm). The following four metrics are used to evaluate the classification performance:

(5) $$accuracy=\frac{\left|TP\right|+\left|TN\right|}{\left|P\right|+\left|N\right|},$$

(6) $$precision=\frac{\left|TP\right|}{\left|TP\right|+\left|FP\right|},$$

(7) $$recall=\frac{\left|TP\right|}{\left|TP\right|+\left|FN\right|},$$

(8) $$F1-Score=\frac{2\times precision\times recall}{precision+recall}.$$

Firstly, only SVM was used for classification. RBF was used as the kernel function, and five-fold cross validation was used. Table 6 shows the confusion matrix of SVM classification. Accuracy = 89.6%, precision = 94.4%, recall = 81.5%, and F1-Score = 87.5%.

Then, we used the DT–SVM for classification. Table 7 shows the confusion matrix of the DT–SVM classification.

Accuracy = 97.6%, precision = 97.2%, recall = 98.3%, and F1-Score = 97.8%. It can be seen that the designed Decision Tree improves the recognition accuracy of daily-life activities significantly, from 81.5% to 98.3%. The proposed school violence detecting algorithm also has better performance than existing violence detecting algorithms (e.g., 96.50% in [16] and 91% in [18]).

## 7. Discussions and Conclusions

School violence is a common social problem that does harm to teenagers. Fortunately, there are now several methods that can detect school violence, such as methods that use movement sensors and those that use cameras. We have used movement sensors to detect school violence in our previous work, but in this paper, we chose another method—one that uses cameras in case the movement sensors are removed by the bullies. The camera was used to take pictures of the monitoring area and detect moving targets. We proposed a circumscribed rectangular frame integrating method to optimize the detected foreground target. Then, the rectangular frame features and optical-flow features were extracted to describe the differences between school violence and daily-life activities. Relief-F and Wrapper were used to reduce feature dimensionality. Then, a DT–SVM classifier was built for classification. The accuracy reached 97.6%, and the precision reached 97.2%. By analyzing the simulation results, we determined that images with great changes in light and shadow and violent actions with slight amplitudes are easily misclassified. This work shows promise for campus violence monitoring. In future work, we intend to involve more complex activities, such as group bullying/fighting and more complex scenes with both nearby and distant objects, and improve the recognition performance of the misclassified samples.

## Figures and Tables

**Table 1:** Numbering the features.

Feature	Number
Max width of CRF(s) ^1^	(1)
Max width variation of CRF(s)	(2)
Max height of CRF(s)	(3)
Max height variation of CRF(s)	(4)
Max area of CRF(s)	(5)
Max area variation of CRF(s)	(6)
Max aspect ratio of CRF(s)	(7)
Max aspect ratio variation of CRF(s)	(8)
Max centroid distance of CRF(s)	(9)
Max centroid distance variation of CRF(s)	(10)
Sum of areas of detected targets	(11)
Max area of detected targets	(12)
State of detected targets	(13)
Mean of optical flow	(14)

^1^ CRF(s): circumscribed rectangular frame(s).

**Table 2:** Accuracy variation during the “backwards” procedure.

Removed Feature(s)	Accuracy (%)	Action
None	89.15	-
(1)	89.02 ↓	Retain (1)
(2)	89.03 ↓	Retain (2)
(3)	88.82 ↓	Retain (3)
(4)	89.05 ↓	Retain (4)
(5)	89.12 ↓	Retain (5)
(6)	89.12 ↓	Retain (6)
(7)	88.60 ↓	Retain (7)
(8)	89.22 ↑	Remove (8)
(8), (9)	89.42 ↑	Remove (9)
(8), (9), (10)	89.35 ↓	Retain (10)
(8), (9), (11)	88.94 ↓	Retain (11)
(8), (9), (12)	89.42 =	Try (12) later
(8), (9), (12), (13)	87.72 ↓	Retain (13)
(8), (9), (12), (14)	89.62 ↑	Remove (14)
(8), (9), (14)	89.52 ↓	Remove (12)

**Table 3:** Accuracy variation during the “forwards” procedure.

Added Feature(s)	Accuracy (%)	Action
None	89.62	-
(8)	89.48 ↓	Remove (8)
(9)	88.86 ↓	Remove (9)
(12)	89.52 ↓	Remove (12)
(14)	89.42 ↓	Remove (14)

**Table 4:** Comparison of the four kernel functions.

Kernel Function	Accuracy (%)
Linear	86.2
Polynomial	86.7
RBF	89.2
Sigmoid	78.1

**Table 5:** Selected features for DT (Decision Tree) and their corresponding thresholds (unit: pixel^2^ for area, and pixel for the others).

Feature	Threshold	Classified As
Max area of detected targets	> 40,000	School violence
Max area variation of CRF(s) ^1^	> 50,000	School violence
Sum of areas of detected targets	< 6000	Daily-life activities
Number of CRF(s)	0	Daily-life activities
Max centroid distance of CRF(s)	> 300	Daily-life activities
Max width of CRF(s)	< 50	Daily-life activities
Max area of CRF(s)	< 10,000	Daily-life activities
Max centroid distance variation of CRF(s)	> 400	Daily-life activities
State of targets	= 1	Daily-life activities
Mean of optical flow	> 2000	Daily-life activities

^1^ CRF(s): circumscribed rectangular frame(s).

**Table 6:** Confusion matrix of SVM (Support Vector Machine) classification.

Classified As	School Violence	Daily-life Activities
School violence	96.1%	3.9%
Daily-life activities	18.5%	81.5%

**Table 7:** Confusion matrix of the DT–SVM (Decision Tree – Support Vector Machine) classification.

Classified As	School Violence	Daily-life Activities
School violence	96.8%	3.2%
Daily-life activities	1.7%	98.3%
